# FAM225A promotes sorafenib resistance in hepatocarcinoma cells through modulating miR-130a-5p–CCNG1 interaction network

**DOI:** 10.1042/BSR20202054

**Published:** 2020-12-16

**Authors:** Yan-Tong Liu, Guo-Qing Liu, Jing-Min Huang

**Affiliations:** 1 School of Basic Medical Sciences, Xi’an Medical University, Xi’an, Shaanxi, 710021, China; 2 Department of Surgical Oncology, Qinghai Provincial People’s Hospital, Xining, Qinghai, 810006, China

**Keywords:** CCNG1, FAM225A, hepatocellular carcinoma, miR-130a-5p, sorafenib resistant

## Abstract

Chemotherapy resistance is still a key hurdle in current hepatocellular carcinoma (HCC) treatment. Therefore, clarifying the molecular mechanisms contributing to this acquired resistance is urgent for the effective treatment of liver cancer. In this research, we observed that lncRNA FAM225A expression is dramatically up-regulated not only in HCC tissues and cell lines but also in sorafenib-resistant HepG2/SOR cells. Moreover, FAM225A knockdown significantly weakened HepG2/SOR cells resistance to sorafenib treatment by MTT (3-(4,5-Dimethylthiazol-2-yl)-2,5-diphenyltetrazolium bromide) assay. Similar results were obtained from the tumor xenograft model in mice. Further mechanistic researches revealed that the direct interaction between FAM225A and miR-130a-5p, while miR-130a-5p negatively modulated Cyclin G1 (CCNG1) expression by targeting 3′UTR of CCNG1. MiR-130a-5p inhibition or CCNG1 overexpression could partially offset FAM225A knockdown-induced increased viability of HepG2/SOR cells in response to sorafenib challenge. Collectively, our findings provide evidence that FAM225A/miR-130a-5p/CCNG1 interaction network regulates the resistance of HCC cells to sorafenib treatment and could supply a possible strategy for restoring sorafenib sensitivity in HCC therapy.

## Introduction

Hepatoma, a frequent malignancy taken place in liver, is one of the leading causes of cancer mortality in globe [1,2]. Most of hepatocellular carcinoma (HCC) patients being diagnosed at advanced stage owing to the absence of early symptoms. Sorafenib, affecting multiple tumor signaling pathways including those participating in cell proliferation, apoptosis and as well as angiogenesis [3,4], was warranted by FDA for the only recommended therapy of advanced HCC patients in 2007. Despite sorafenib has encouraging effects on improving overall survival, the clinical application remains largely restricted by the acquired sorafenib resistance [5,6]. Therefore, elucidating the driving force of resisting to sorafenib treatment in HCC patients will be helpful to develop new strategies for conquering this resistance.

Accumulating evidence has declared that long-chain non-coding RNAs (lncRNAs) with a size exceeding 200 nucleotides, are essential mediators regulating the progression of different kinds of cancer and become promising therapeutic targets [7,8]. Owing to their aberrant expression and high tissue specificity, lncRNAs have become good biomarkers and prognostic factors in different cancers [9–11]. These lncRNAs act as oncogenes or tumor suppressors via a series of mechanisms dependent on chromatin modification or interacting with other RNAs in HCC development [12,13]. LncRNA FAL1 could sponge to miR-1236, thus leading to enhanced abilities of cell proliferation and metastasis in HCC [14]. On the contrary, TSLNC8 could inhibit tumor progression in HCC via a post-transcriptional modification-dependent mechanism [15]. FAM225A was a newly identified lncRNA and functioned as an oncogene that promoted nasopharyngeal carcinoma cell viability and invasion by trapping miR-590-3p and miR-1275, indicating a functional role in tumorigenesis and metastasis [16]. However, the expression and functions of FAM225A in HCC has not been reported.

Recently, several lncRNAs have been identified as regulators in chemotherapeutic resistance. For example, HCC-associated lncRNA termed as HANR suppresses doxorubicin sensitivity of HCC cells by binding to GSKIP for regulating GSK3β phosphorylation [17]. LncRNA NR2F1-AS1 level is increased and NR2F1-AS1 overexpression attenuates oxaliplatin sensitivity via modulating miR-363-ABCC1 axis in HCC [18]. The enhanced expression of lncRNA PDIA3P1 confer chemoresistance of HCC by serving as a microRNA sponge to up-regulate TRAF6 expression and amplify NF-κB signaling [19].

In this investigation, we observed high levels of lncRNA FAM225A expression both in HCC tissues and in sorafenib-insensitive HCC cells. Additionally, dysregulation of FAM225A and miR-130a-5p remarkably affected hepatoma cell sensitivity to sorafenib via targeting Cyclin G1 (CCNG1), which might provide us with more potential therapeutic strategies for chemosensitization of HCC cells to sorafenib.

## Materials and methods

### Patient samples

Thirty HCC and corresponding normal tissue samples were obtained from Qinghai Provincial People’s Hospital. Both clinical tissues collection and the procedures in the research were permited by the Ethics Committee of Qinghai Provincial People’s Hospital. All patients involved in the study signed their informed consent.

### Cell lines and reagents

We purchased four human hepatoma cell lines including SK-hep1, HepG2, Huh7 and HCCLM3 and as well as normal control cell line LO2 from the American Type Culture Collection (Manassas, VA, U.S.A.). To establish sorafenib-resistant cells, HepG2 cells were challenged with different concentrations of sorafenib. Six months later, HepG2-SOR cells which referred to HepG2 cells resisted to sorafenib was generated. Cells were incubated in DMEM (HyClone) containing with 10% FBS (Gibco) at 37°C in 5% CO_2_ incubation. The sorafenib-resistant cells were continuously maintained by culturing them in medium containing sorafenib at a dose of 10 μM.

Mimics, inhibitors and negative control oligonucleotides for miR-130a-5p were from Sangon Biotech company (Shanghai, China). SiRNAs and plasmids overexpressing CCNG1 were purchased from Genechem company (Shanghai, China). Cell transfections were performed by using Lipofectamine 2000 reagent (Invitrogen). Sequence of miR mimics and inhibitors were shown in Table 1.

**Table 1:** The sequences of miRNA mimic, inhibitor used in the present study

Name	Sequence
miR-130a-5p mimic	5′-UUCACAUUGUGCUACUGUCUGC-3′
NC mimic	5′-UUCUCCGAACGUGUCACGUTT-3′
miR-130a-5p inhibitor	5′-CAGACAGTAGCACAATGTGA-3′
NC inhibitor	5′-UCACAACCUCCUAGAAAGAGUAGA-3′

### MTT assay

A total of 3 × 10^3^ cells were planted into each well of a 96-well plate and grown under various concentrations of sorafenib. Viable cells were measured by MTT (3-(4,5-Dimethylthiazol-2-yl)-2,5-diphenyltetrazolium bromide) assay as previously described [20].

### Quantitative real-time PCR

Total RNAs were collected from HCC tissues, adjacent normal tissues and cells with the use of TRIzol reagent (Invitrogen) and reverse transcribed into cDNA by PrimeScript™ Kit (Takara, Dalian, China). The primers used were as follows: for FAM225A, 5′-CCTGCGTCTTCTGCCACTGC-3′ (forward) and 5′-GGAGGACAGCAAGCACGACAG-3′ (reverse); for miR-130a-5p, 5′-GCCGCTCTTTTCACATTGTGCTACT-3′ (forward) (Universal miRNA reverse primer: proprietary, B532451-0020); for CCNG1, 5′-GTTACCGCTGAGGAGCTGCAGTC-3′ (forward) and 5′-GCAGCCATCCTGGATGGATTCAG-3′ (reverse); GAPDH, 5′-GGGAGCCAAAAGGGTCAT-3′ (forward) and 5′-GAGTCCTTCCAC GATACCAA-3′ (reverse); U6, 5′-AGAGAAGATTAGCATGGCCCCTG-3′ (forward) and 5′-ATCCAGTGCAGGGTCCGAGG-3′ (reverse). The target gene expression was first normalized to the levels of GAPDH (for mRNA) or U6 (for miRNA), the calculated results were then normalized to the control groups using the comparative threshold cycle (2^−ΔΔ*C*_t_^) method. Three biological repeats were performed for each test.

### Dual-luciferase reporter assay

The luciferase reporter plasmids encoding wild-type or mutant 3′UTR of FAM225A and CCNG1 were constructed by GenePharma (Shanghai, China). Then the reporter plasmids including wt (mut) 3′-UTR of FAM225A or wt (mut) CCNG1 and miR-130a-5p or control mimics or control were co-transfected into cells. The luciferase activity was measured at 48 h after transfection by a Luciferase Reporter System (Beyotime).

### RNA-binding protein immunoprecipitation

RIP was performed using a Magna RIP RNA-Binding Protein Immunoprecipitation kit (Millipore, Bedford, MA) according to the manufacturer’s instructions. Briefly, 2 × 10^6^ cell lysates were incubated with magnetic beads conjugated with negative control normal mouse IgG or human anti-Ago2 antibody (Millipore). The immunoprecipitated RNAs were then extracted and detected by qRT-PCR to confirm the enrichment of binding targets and the products were then subjected to agrose gel electrophoresis.

### Western blotting

Proteins were extracted from cells or tissues using a commercial protein extraction kit. Then, SDS/PAGE was performed according to a previous method [21]. Anti-CCNG1 primary antibody (Sigma) and horseradish peroxidase (HRP)-conjugated secondary antibody (1:10000) was used to incubate with the membrane. The NIH ImageJ software was used to visualize the results with β-actin served as an internal control. The obtained images were converted into 8-bit format in order to perform uncalibrated optical density (OD). After conversion, the background was subtracted through the rolling ball radius method. Each band was individually selected and circumscribed with the rectangular ROI selection, followed by quantification of peak area of obtained histograms. Data were acquired as mean gray values.

### Animal tumor xenograft model

The study was performed at Qinghai Provincial People’s Hospital. All experiments were performed following the Guidelines of Animal Use and Care Committee of the Qinghai Provincial People’s Hospital (Qinghai, China), and the present study was approved by the Institutional Review Board of Qinghai Provincial People’s Hospital (number: XJTULAC20181352). The male nude mice (5–6 weeks old) maintained in the animal facility of Qinghai Provincial People’s Hospital maintained at room temperature (22 ± 1°C) with a 12/12-h light/dark cycle and access to food and water *ad libitum*. Totally, twenty male nude mice (5–6 weeks old, five mice per group) were received subcutaneous injections of 1 × 10^6^ HepG2-SOR cells in 200 μl phosphate buffer saline (PBS) transfected with shNC or shFAM225A in the right flank area. The tumor size was monitored at an interval of 3 days. One-week post injection, sorafenib at a dose of 10 mg/kg or the vehicle control were given to mice orally once every day. At the indicated days after inoculation, the mice were anesthetized by intramuscular injection of 50 mg/kg ketamin mixed with 5 mg/kg xylazine. Then, the mice were killed by cervical dislocation, and xenografts were weighted.

### Statistical analysis

All results were displayed as mean ± standard error and statistical analysis were applied by using GraphPad Prism 7.0 (GraphPad Software). For comparing two or more groups, the Student’s *t* test or one-way analysis of variance was applied. A probability level of less than 0.05 was supposed to be statistically significant.

## Results

### HCC tissues and cell lines displayed high levels of FAM225A

**Figure 1:**
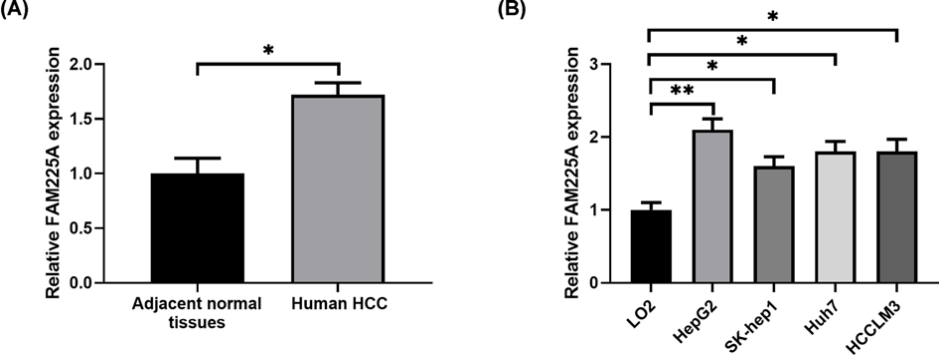
Relative levels of FAM225A in HCC tissues and cell lines (**A**) FAM225A expression in the human HCC and adjacent normal tissues detected by RT-PCR. (**B**) FAM225A expression levels in HCC cell lines and normal liver cell line LO2. *P<0.05, **P<0.01, compared with adjacent normal tissues or LO2.

In order to explore whether FAM225A was involved in HCC progression, we first measured the FAM225A expression in 30 human HCC samples and corresponding non-tumor controls using qRT-PCR assay. The results showed that HCC samples exhibited obviously higher FAM225A level than normal tissues (Figure 1A, *P*<0.05). Consistent with this observation, the levels of FAM225A in four HCC cell lines also displayed remarkable increases compared with control LO2 cells (Figure 1B, *P*<0.05). Collectively, these data demonstrated that FAM225A expression was significantly increased in HCC tissues and cells, suggesting that the FAM225A may be associated with HCC development.

### FAM225A level is increased in HCC cells resistant to sorafenib

**Figure 2:**
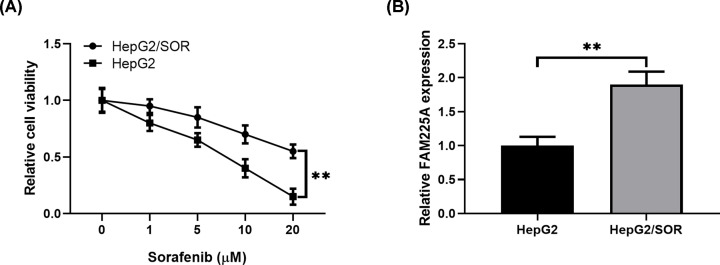
FAM225A is up-regulated in sorafenib-resistant HCC cells (**A**) MTT assay was performed to analyze the sorafenib resistance of HepG2 cells and HepG2/SOR (sorafenib-resistant) cells treated with various concentrations of sorafenib. (**B**) Relative levels of FAM225A in HepG2 and HepG2/SOR cells was measured by RT-qPCR analysis. **P<0.01, compared with HepG2.

Next, to further investigate whether FAM225A level was correlated with the sensitivity of HCC cells to sorafenib treatment, the HepG2 cells insensitive to sorafenib (named as HepG2/SOR) were generated from HepG2. MTT results showed that sorafenib displayed a remarkably poorer suppression effect on HepG2/SOR cells than HepG2 cells (Figure 2A, *P*<0.05). Moreover, HepG2/SOR exhibited higher levels of FAM225A expression than those in HepG2 cells (Figure 2B, *P*<0.05).

### FAM225A knockdown strengthens cell sensitivity to sorafenib in HCC

**Figure 3:**
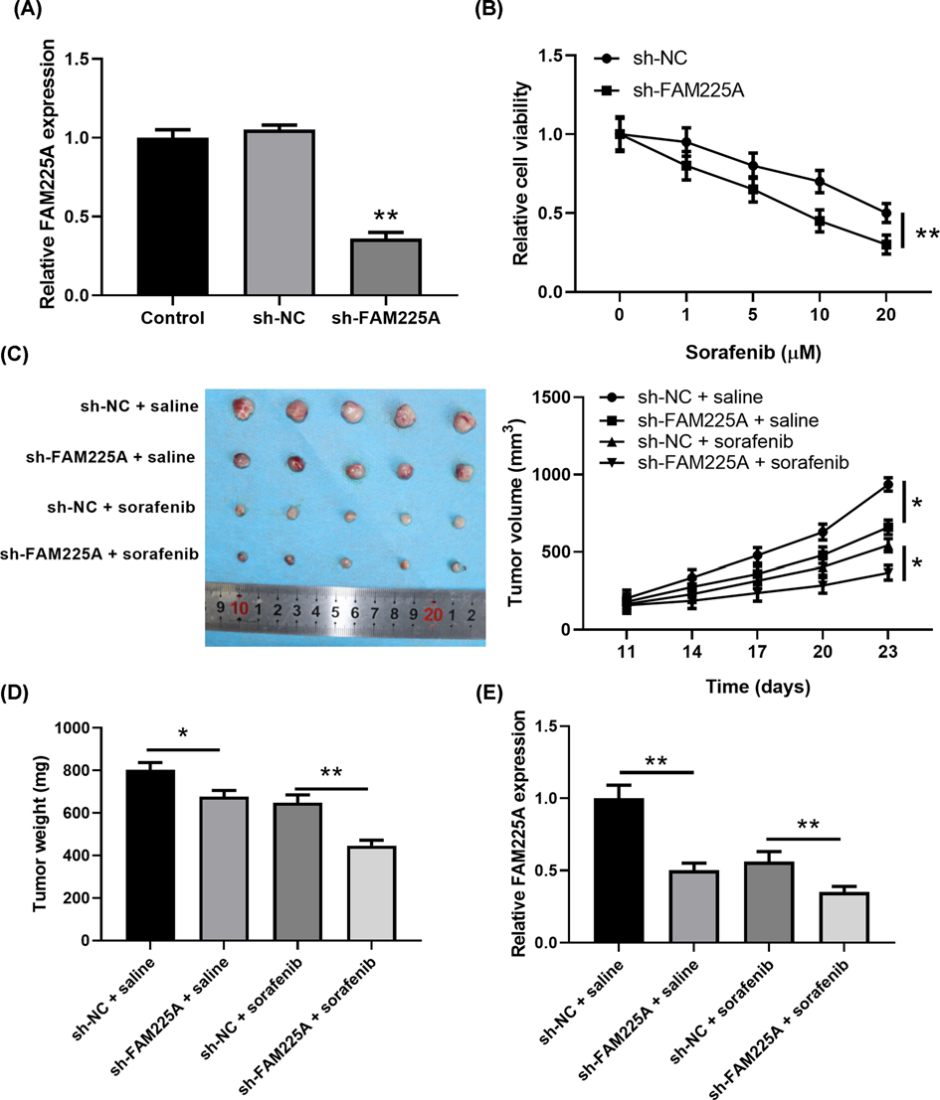
Knockdown of FAM225A attenuates sorafenib resistance *in vitro* and *in vivo* (**A**) Knockdown efficacy was determined by RT-qPCR analysis. (**B**) The effect of FAM225A knockdown on sorafenib resistance of HepG2/SOR cells was analyzed by MTT assay. (**C**) The tumor volume was measured at 11, 14, 17, 20 and 23 days, and the tumor growth curves were plotted. (**D**) Twenty days after cell inoculation, the tumors were excised and weighed. (**E**) Relative levels of FAM225A in the tumor tissues was measured by RT-qPCR analysis. *P<0.05, **P<0.01, compared with sh-NC+saline or sh-NC+sob.

To deeply dissect the exact function of FAM225A in sorafenib tolerance, HepG2/SOR cells silencing FAM225A expression were constructed with transfection of a small interfering RNA against FAM225A. The qPCR assay was performed to testify the transfection efficiency and our results showed that the expression of FAM225A was significantly down-regulated (Figure 3A, *P*<0.05). MTT results showed that HepG2/SOR cells silencing FAM225A expression exhibited an increased sensitivity to sorafenib challenge (Figure 3B, *P*<0.05). In order to verify the inhibitory effects of FAM225A on sorafenib sensitivity, we constructed a tumor xenograft model by subcutaneously injecting HepG2/SOR cells stably knockdown of FAM225A to nude mice. *In vivo* experiment showed that administration of sorafenib markedly reduced the mean tumor volume and weight, and slow tumor growth compared with that in control group. Moreover, shFAM225A combined with sorafenib treatment displayed the most remarkable inhibitory effect on tumor volume, weight and growth (Figure 3C,D, *P*<0.05). The effects of FAM225A knockdown in xenograft tumor were verified by qRT-PCR analysis (Figure 3E).

### FAM225A directly interacts with miR-130a-5p

**Figure 4:**
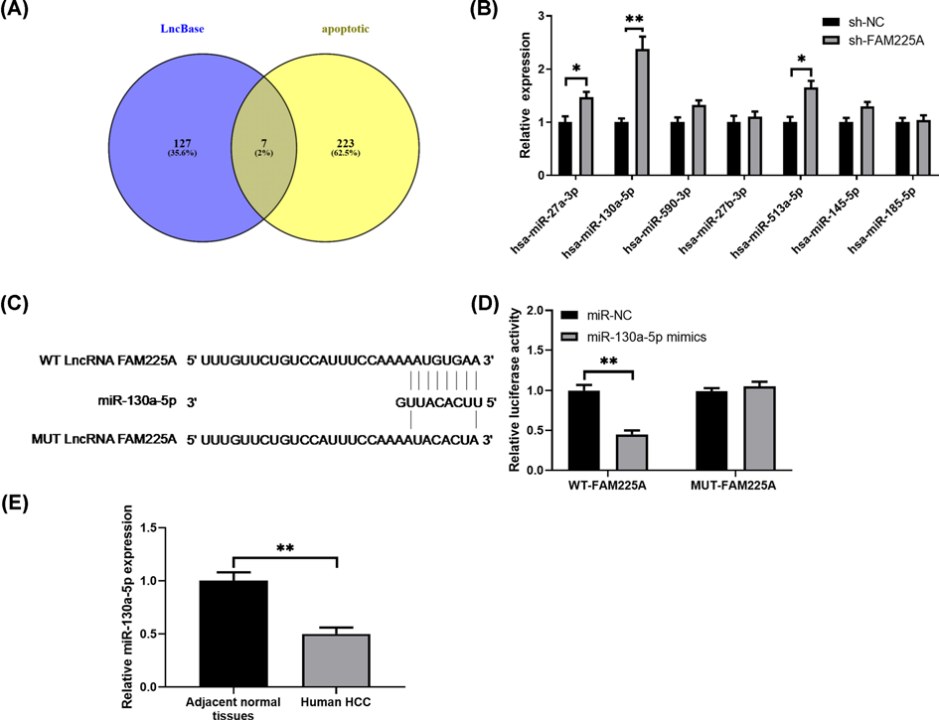
FAM225A directly interacts with miR-130a-5p in HCC cells (**A**) Bioinformatics analysis revealed the predicted binding sites between FAM225A and miR-130a-5p. (**B**) HepG2 cells were co-transfected with FAM225A-WT or FAM225A-MUT, together with miR-130a-5p or miR-NC, and after 48 h, the luciferase activities were measured. (**C**) Sequence alignment showing possible interactions between miR-130a-5p and wild-type (Wt-FAM225A) or mutated (Mut-FAM225A) 3′-UTR of FAM225A. (**D**) Luciferase reporter activities of cells co-transfected with plasmid containing wild-type or mutated FAM225A 3′-UTR and miR-130a-5p mimics. Cells co-transfected with control miRNA (miR-NC) were used as control. *P<0.05, compared with miR-NC. (**E**) Relative levels of miRNA in adjacent tissues and HCC. *P<0.05, **P<0.01, comapred with sh-NC,miR-NC or adjacent tissues.

In an effort to explore the downstream regulation mechanisms of FAM225A, lncBase and GO analysis was conducted to predict the potential miRNA targets of FAM225A and seven miRNAs were found (Figure 4A). Using qRT-PCR analysis, we found that FAM225A ablation could elevate the level of miR-27a-3p, miR-130a-5p and miR-513a-5p (Figure 4B). Among these three miRNAs, miR-130a-5p changed most dramatically. Therefore, we mainly focused on miR-130a-5p in the following experiments. As shown in Figure 4C, miR-130a-5p has a potential complementary binding sequence to FAM225A. To confirm this prediction, luciferase reporter assay examined that miR-130a-5p mimic efficiently reduced the luciferase activity of WT-FAM225A but not mut-FAM225A in HepG2 cells (Figure 4D). In addition, the levels of miR-130a-5p were remarkably declined in HCC tissues (Figure 4E).

### MiR-130a-5p inhibitor offset the enhanced sorafenib sensitivity induced by FAM225A knockdown

**Figure 5:**
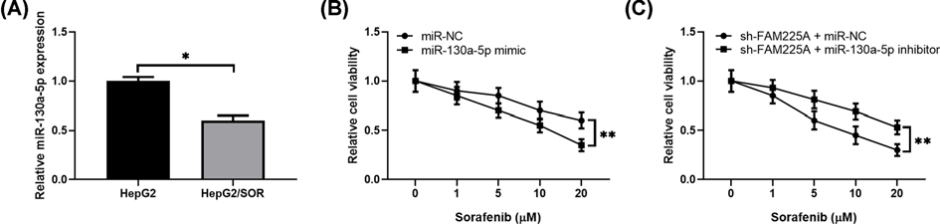
MiR-130a-5p inhibitor abrogates the effects of FAM225A knockdown on sorafenib resistance in HCC cells (**A**) Relative levels of miR-130a-5p in HepG2/SOR (sorafenib-resistant) and HepG2 cells were measured by RT-qPCR analysis. (**B**) The effect of miR-130a-5p overexpression on sorafenib resistance of HepG2/SOR cells was analyzed by MTT assay. (**C**) The effect of miR-130a-5p inhibitor on sorafenib resistance of sh-FAM225A-transfected HepG2/SOR cells was analyzed by MTT assay. *P<0.05, **P<0.01, compared with HepG2, miR-NC or sh-FAM225A+miR-NC.

Next, we explored the effects of miR-130a-5p dysregulation on sorafenib sensitivity in HCC. First, we noticed that miR-130a-5p expression level was obviously lower in HepG2/SOR cells than that in control cells (Figure 5A). MTT results showed that HepG2/SOR cells transfection with miR-130a-5p mimic significantly reduced sorafenib resistance compared with those cells transfection with miR-NC (Figure 5B). To further clarify whether FAM225A knockdown alleviates sorafenib resistance in HCC by regulating miR-130a-5p, MTT assay was performed in HepG2/SOR cells co-transfection with shFAM225A and miR-130a-5p inhibitor. Our data demonstrated that miR-130a-5p inhibitor partially abolished the increased sensitivity of HepG2/SOR cells to sorafenib induced by FAM225A knockdown (Figure 5C).

### FAM225A/miR-130a-5p axis regulates sorafenib resistance of HCC cells by targeting CCNG1

**Figure 6:**
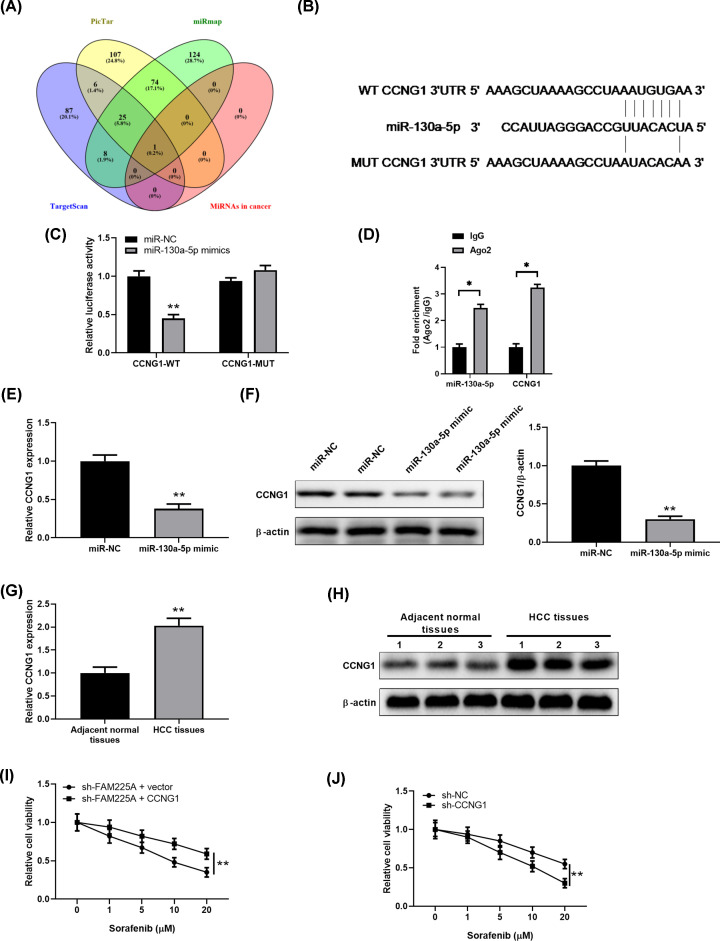
MiR-130a-5p targeted CCNG1 to modulate its expression in HCC cells (**A,B**) Bioinformatics analysis revealed the predicted binding sites between CCNG1 and miR-130a-5p. (**C**) HepG2 cells were co-transfected with CCNG1-WT or CCNG1-MUT, together with miR-130a-5p mimic or miR-NC, and after 48 h, the luciferase activities were measured. (**D**) Immunoprecipitants of CCNG1 and miR-130a-5p in IgG and Ago2. *P<0.05, compared with IgG. (**E,F**) miR-130a-5p mimics decreased the mRNA and protein expression of CCNG1 in HepG2 cells compared with negative control. (**G**) Relative levels of CCNG1 was significantly down-regulated in HCC tissues compared with adjacent normal tissues by quantitative real-time PCR (*n*=30). (**H**) Representative Western blot results of CCNG1 protein levels in in HCC tissues and adjacent normal tissues (*n*=30). (**I**) MTT assay was performed to analyze the sorafenib resistance of HepG2/SOR (sorafenib-resistant) cells transfected with si-CCNG1 or siRNA Con. (**J**) MTT assay was performed to analyze the sorafenib resistance of HepG2/SOR cells transfected with si-FAM225A, or along with CCNG1. **P<0.01, compared with miR-NC, adjacent normal tissues or sh-FAM225A.

The important role of miRNAs in the regulating drug resistance caused us to search for the target gene of miR-130a-5p [22]. Bioinformatics algorithms including TargetScan, miRWalk, miRDB, and MicroRNAs in cancer (KEGG) were employed and CCNG1 was obtained (Figure 6A). As shown in Figure 6B, miR-130a-5p has a potential complementary binding sequence to CCNG1 (1308-1330). Luciferase reporter assay confirmed the direct interaction between miR-130a-5p and CCNG1 as miR-130a-5p mimic dramatically suppressed the luciferase activity of reporter containing WT sequence of CCNG1 3′UTR (Figure 6C). To further validate the interaction between miR-130a-5p and CCNG1, we performed RNA-binding protein immunoprecipitation. In the binding RNAs pulled-down by AGO2 antibody, the level of CCNG1 mRNA was significantly decreased when transfected with miR-130a-5p mimics (Figure 6D). Next, the regulation role of miR-130a-5p in CCNG1 level was assessed. CCNG1 mRNA and protein expression were dramatically reduced in HepG2 transfection with miR-130a-5p mimic (Figure 6E,F). Interestingly, qRT-PCR and Western blot analysis results displayed notably higher levels of CCNG1 expression in HCC tissues than controls (Figure 6G,H), suggesting CCNG1 gene was modulated by FAM225A/miR-130a-5p axis. Consistently, HepG2/SOR cells silencing CCNG1 displayed a declined resistance to sorafenib compared with control (Figure 6I). Similarly, HepG2/SOR cells transfected with shFAM225A in combination with CCNG1-expressing vector partially abrogated the enhanced sensitivity to sorafenib induced by FAM225A knockdown (Figure 6J). Collectively, our findings provide proofs that FAM225A promoting sorafenib resistance in HCC cells is dependent on the miR-130a-5p/CCNG1 axis.

## Discussion

Although Sorafenib is the standard of care and the only recommended treatment for treating advanced HCC, some patients ultimately develop resistance to this drug in clinic settings. Hence, it is of great value to explore the underlying force driving cancer cells insensitive to sorafenib and seek out novel targeting molecules for conquering this kind of resistance. In the current research, we identified lncFAM225A level to be highly increased in HCC, and as well as in sorafenib-resistant cells. *In vitro* cell experiments and *in vivo* animal data revealed that knockdown of FAM225A led to enhanced sorafenib sensitivity. Further mechanistic explorations disclosed that FAM225A promoted sorafenib resistance by competitively binding to miR-130a-5p, leading to the up-regulation of CCNG1. Therefore, FAM225A was testified to act as a vital role in sorafenib resistance in HCC.

FAM225A was found to be one of the most highly expressed lncRNAs in nasopharyngeal carcinoma by a microarray analysis. FAM225A could regulate ITGB3 expression by sponging miR-590-3p and miR-1275 and resulted in tumorigenesis and metastasis, indicating its oncogenic role in tumor development [16]. In accordance with this, we also found that FAM225A expression was significantly up-regulated in both tissues and cell lines from HCC, suggesting that FAM225A served as an oncogene in HCC. Moreover, compared with HepG2 cells, HepG2/SOR cells displayed higher levels of FAM225A. The results based on cell lines and animals revealed that FAM225A ablation significantly increased sorafenib sensitivity of HCC. These data suggested that the elevated expression of FAM225A might be responsible for sorafenib resistance in HCC. However, it is noteworthy that the clinical sample size in our study was relatively small and the studied participants were limited to Chinese individuals from Northwest China. Further studies are needed to confirm the results in larger sample size.

Recently, accumulating evidence have shown that miRNAs are important regulators in drug resistance in a broad spectrum of cancers, offering novel molecules for diagnosis and new strategies for the treatment of HCC [23–26]. Our results further demonstrated that FAM225A served as a competing endogenous RNA (ceRNA) for sponging miR-130a-5p. Several studies indicated that miR-130a-5p exerted a suppressive role in tumor development. For example, miR-130a-5p inhibited tumor invasiveness and development in esophageal squamous cell carcinoma via negative regulation of ZEB1 [27]. In another study, the lower expression of miR-130a-5p resulted in a reduced suppression of miR-130a-5p on HMGB2, thus contributing to glioma growth and metastasis [28]. Our results also revealed that FAM225A directly interacted with miR-130a-5p by luciferase reporter assay, and miR-130a-5p expression was obviously lower in HCC tissues and in sorafenib-resistant cells, confirming the role of miR-130a-5p acting as a tumor suppressor. In addition, FAM225A knockdown caused an augment of miR-130a-5p expression, and miR-130a-5p inhibitor abrogated the enhanced sensitivity of HCC cells to sorafenib treatment caused by FAM225A deletion. Therefore, our observations uncovered the important functions of FAM225A and miR-130a-5p in sorafenib resistance in HCC.

Next, with bioinformatics tools, CCNG1 was predicted and testified to be a direct target gene of miR-130a-5p in HCC cell lines. CCNG1 dysregulation is described in different kinds of cancer [29–32], suggesting its necessary role in tumor development. Some miRNAs has been reported to interact with CCNG1 to affect tumor progression and chemoresistance [33]. For example, miR-27b could enhance the sensitivity of gastric cancer cells to several chemotherapeutic drugs by suppressing CCNG1, suggesting that CCNG1 may contribute to chemoresistance [22,34]. Up to now, we first reported that decreased miR-130a-5p expression can result in increased level of CCNG1, thus contributing to the resistance to sorafenib in HCC.

## Conclusions

In summary, lncRNA FAM225A played an essential role in sorafenib-resistant HCC cells, up-regulation of FAM225A could exacerbate drug resistance as a ceRNA by serving as a sponge to damage miR-130a-5p-dependent CCNG1 down-regulation. These results supplied with a novel insight into the molecular mechanism of chemoresistance and indicated targeting the FAM225A/miR-130a-5p/CCNG1 regulatory axis might offer a new strategy for treating sorafenib resistance in HCC.

## Data Availability

The datasets used and/or analyzed during the current study are available from the corresponding author on reasonable request.

## Abbreviations

AGO2: Argonaute 2
CCNG1: Cyclin G1
ceRNA: competing endogenous RNA
FAM225A: Family With Sequence Similarity 225 Member A
GSKIP: GSK3β interacting protein
GSK3β: glycogen synthase kinase-3beta
HCC: hepatocellular carcinoma
ITGB3: integrin β3
KEGG: Kyoto Encyclopedia of Genes and Genomes
lncRNA: long non-coding RNA
MTT: 3-(4,5-Dimethylthiazol-2-yl)-2,5-diphenyltetrazolium bromide
qPCR: quantitative real-time PCR
RIP: RNA immunoprecipitation
ROI: region of interest
